# From Optical Transitions Through Morphology Changes to Charge Transport: A P3HT Benchmark Study Using the Critical Point Model

**DOI:** 10.3390/polym18212673

**Published:** 2026-10-28

**Authors:** Tal Elbaz, Chithira Pulickalputhenpura Rajan, Rafi Shikler

**Affiliations:** 1 School of Electrical & Computer Engineering, Ben-Gurion University of the Negev, Beer-Sheva 8410501, Israel; chithira.pr@presidencyuniversity.in (C.P.R.); rshikler@bgu.ac.il (R.S.); 2 Department of Physics, Presidency University, Bengaluru 560064, India; 3 Ilse Katz Institute for Nanoscale Science and Technology, Ben-Gurion University of the Negev, Beer-Sheva 8410501, Israel

**Keywords:** organic thin-film transistors (OTFTs), P3HT, spectroscopic ellipsometry, critical point model, charge transport, morphology

## Abstract

Organic and polymer semiconductors are central to modern electronic and optoelectronic technologies, including organic thin-film transistors (OTFTs), solar cells, and bioelectronic devices. Controlling thin-film morphology is critical for optimizing the performance of organic electronic devices. However, the spin-coating process provides limited morphological control, and conventional characterization tools such as X-ray diffraction (XRD) and atomic force microscopy (AFM) are often insensitive to subtle structural changes that strongly influence charge transport. In this work, we demonstrate that spectroscopic ellipsometry (SE) combined with critical point (CP) analysis enables rapid, non-destructive assessment of microstructural evolution in regioregular poly(3-hexylthiophene) (rr-P3HT) thin films during thermal annealing. OTFTs based on P3HT were selected as a benchmark system due to their simple device architecture and well-defined structure–property relationships. The CP model provides physically meaningful optical parameters (transition energy, amplitude, broadening, and dimensionality) that correlate with electrical performance. Our results reveal a distinct shift in dimensionality of the primary optical transition upon annealing, indicating increased molecular ordering and reduced energetic disorder. These optically derived descriptors complement conventional structural probes and correlate with the observed improvements in charge transport. These findings offer a practical, non-destructive materials-focused approach for early-stage evaluation and process monitoring in organic electronics.

## 1. Introduction

Over the last decade, organic-based electronic and optoelectronic devices have become increasingly popular. Specifically, organic thin-film transistors (OTFTs) are attracting growing interest due to their potential for low-cost, lightweight, flexible, and large-area electronic applications. These characteristics make OTFTs ideal candidates for electronic applications such as flat-panel displays, sensors, and medical sensing applications [1,2,3,4,5,6]. A key factor influencing the performance of organic-based devices, including OTFTs, is the morphology of the organic semiconducting active layer. In typical OTFTs, charge transport occurs via hopping between localized states [7,8], making it highly sensitive to structural and energetic disorder [9,10]. While convenient and scalable [6], the commonly used spin coating method for depositing the active organic semiconductor layer often results in unpredictable film morphologies due to its inherent higher degree of disorder [11,12,13]. This morphological disorder significantly impacts charge transport. As a result, the charge carrier mobility and device efficiency are reduced [14,15,16,17]. Therefore, optimizing the morphology of the active layer is essential for achieving reliable and high-performance operation [1,18,19,20,21,22,23,24,25]. Post-deposition thermal annealing is a commonly employed technique for promoting polymer chain reorganization and the formation of crystalline domains, which serve as efficient pathways for charge carriers and facilitate efficient charge transport [18,19,22,26,27,28,29,30,31]. The first step toward understanding how annealing-induced morphological changes affect electrical performance is establishing a correlation between morphological evolution and charge transport. For this purpose, direct characterization techniques, including X-ray diffraction (XRD) and atomic force microscopy (AFM), are frequently employed. However, AFM is limited to surface topography and does not capture bulk morphology, while interpreting XRD data is challenging due to the low crystallinity of the films, regardless of annealing duration. Even when applicable, these methods typically do not yield physical quantities that can be directly correlated with electrical behavior. Hence, indirect methods are often employed to extract parameters related to morphology rather than morphology itself. For instance, spectroscopic ellipsometry (SE) provides information on the real and imaginary components of the dielectric function (permittivity), which, due to its first-order linear response, is sensitive to both morphological structure and electronic performance. Establishing this correlation, a theoretical model capable of bridging morphology and device performance, is highly desirable for advancing organic semiconductor devices. Spano’s H/J-aggregate model offers a theoretical framework for quantitatively assessing interchain coupling and intrachain order through the relative intensity of the 0–0 and 0–1 optical peaks in regioregular poly(3-hexylthiophene) (rr-P3HT). Specifically, the ratio $A_{0-0}/A_{0-1}$ decreases with increasing interchain coupling (*W*) and increases with greater intrachain order and conjugation length. This model provides insights into microscopic ordering and aggregate coherence, serving as indicators of molecular ordering and crystallinity. Higher ratios suggest enhanced lamellar stacking and more efficient charge transport pathways, especially in rr-P3HT films with a dominant 0–0 transition. In weakly interacting H-aggregates such as rr-P3HT, this ratio can be directly related to the exciton bandwidth *W* [16,21,32,33,34,35]. However, applying Spano’s model requires fitting the SE data to a Hamiltonian and detailed knowledge of excitonic coupling parameters, which can limit its accessibility for routine analysis. As a simpler alternative, we propose the application of the CP model developed by Cardona [36]. This model identifies interband transitions in the optical spectra that are directly related to the electronic band structure and joint density of states (JDOS) [37]. Since the JDOS is inherently related to charge transport, the CP model offers a route for linking optical transitions to both electronic performance and morphological evolution. The model yields quantitative parameters, such as the dimensionality of the transition (ranging from 0 to 3), amplitude, critical transition energy, and broadening, that are sensitive to microstructural changes. These parameters can serve as indirect yet informative descriptors to support the optimization of optoelectronic properties. In this study, we will demonstrate how applying CP analysis to the dielectric function derived from SE data enables the extraction of detailed insights into the microstructural evolution of the active-layer films. Understanding how processing parameters translate into functional outcomes, based solely on SE data, could enable process optimization and performance prediction, providing a more detailed correlation. To this end, we fabricated OTFTs based on rr-P3HT using the spin-coating method, followed by thermal annealing at varying durations. The devices were characterized via SE, XRD, and current–voltage (*I*–*V*) measurements to probe their optical, structural, and electrical properties, respectively. Although newer materials with higher mobility and better charge separation performance are available, P3HT was selected as the active layer polymer due to its widespread use, well-established properties, and extensive study in organic electronics [23,24,26,38,39,40]. Due to its deep understanding of structure and optoelectronic properties, P3HT is regarded as a benchmark material, enabling comparison with well-established research, such as the aforementioned work by Spano. Moreover, it is still being actively studied and used for numerous applications, such as perovskite solar cells [41,42,43] and bio-electronic and electrochemical applications [44,45]. The rr-P3HT typically exhibits hole charge carrier transport in the range of $10^{-5}$–$10^{-3}$ cm^2^ V^−1^ s^−1^, depending strongly on processing conditions and resulting morphology [2,8,15,46]. P3HT also displays characteristic excitonic absorption features at 604 nm (0–0), 554 nm (0–1), and 524 nm (0–2), corresponding to transitions from the electronic ground state to excited states [16,21,33]. By correlating the parameters 0–0/0–1 peak area ratio and broadening parameter Γ, with the electrical performance of the devices, we establish a non-destructive framework for monitoring and optimizing organic semiconductor morphology. This approach demonstrates the sensitivity of CP analysis to subtle structural transitions that are often unresolved by conventional techniques, offering potential for early-stage process monitoring in organic electronics manufacturing.

## 2. Materials and Methods

### 2.1. OTFT Fabrication

OTFTs were fabricated on a p-doped, 525 μm thick silicon substrate with a thermally grown 90 nm silicon dioxide (SiO_2_) insulating layer. The substrate also served as the gate electrode. Spiral-patterned, 60 nm thick gold (Au) source and drain contacts with a 1 mm overall diameter (channel width, *W*) and a 10 μm channel length (*L*) were deposited via thermal evaporation. To improve adhesion, a thin titanium (Ti) adhesion layer was deposited between the Au contacts and the SiO_2_ surface. The semiconducting active layer (AL) was formed by spin-coating a rr-P3HT solution in chloroform onto the substrate. Post-deposition, the devices were thermally annealed at 140 °C for varying durations (5, 10, 20, and 30 min) on a preheated hot plate inside a nitrogen-filled glovebox to minimize exposure to oxygen and moisture. A separate device was prepared for each annealing duration, and each device underwent a single annealing treatment at its designated duration, so all samples are independent per annealing condition, thus corresponded to an independently prepared device rather than repeated annealing of the same device. Additionally, P3HT thin films, of the same solution used for the OTFTs, were deposited onto bare SiO_2_/Si substrates using the same spin-coating protocol for separate optical and structural characterization, as depicted in Figure 1a. These films were subjected to the same annealing conditions as the OTFT devices, yielding a film thickness of ∼100 nm (from ellipsometry). A more detailed fabrication process is presented in Section S1 in the Supplementary Materials. A schematic illustration of the fabrication process is shown in Figure 1.

### 2.2. OTFT Characterization

To evaluate the impact of annealing on active layer properties, the fabricated OTFT samples were characterized using three complementary techniques: (i) *I–V* measurements using a source measurement unit (SMU) to determine electrical performance, (ii) XRD to assess film crystallinity and morphology, and (iii) SE to probe optical properties.

#### 2.2.1. Electrical Performance

Electrical performance was evaluated with a Keithley 2636B source-meter unit by recording the OTFT’s output characteristics ($I_{D}$–$V_{D}$) curves at fixed gate voltages of 0, −10, −20, −30, and −40 V. The effective mobility, $\mu_{eff}$, and threshold voltage, $V_{T}$, reported in Table 1, were extracted from the linear regime using the Y-function method, following Kim et al. [47]. The extracted Y-function mobility values are summarized in Section S2.1 and Table S1 of the Supplementary Materials. The linear-regime condition, $|V_{D}\left|\ll \right|\left(V_{G}-V_{T}\right)|$ [46,48], was satisfied by selecting a sufficiently small drain voltage. Here, *W* and *L* denote the channel width and length, respectively, and $C_{ox}$ is the capacitance per unit area of the SiO_2_ gate dielectric, calculated to be $38.4\;\mathrm{nF}/{\mathrm{cm}}^{2}$.

#### 2.2.2. Morphological and Structural Characterization

The structural ordering of the P3HT thin films as a function of annealing time was investigated via XRD measurements using a multi-purpose diffractometer with Cu Kα radiation (λ=1.540 Å). XRD patterns were recorded in a θ/2θ scan geometry over a 2θ range of $4^{\circ }$ to $40^{\circ }$, targeting characteristic diffraction peaks associated with crystalline domains within the polymer matrix. The (100) reflection was fitted for each annealing time to obtain the peak position, full width at half maximum (FWHM), and integrated area, as depicted in Section S2.3.1 of the Supplementary Materials. As a symmetric θ/2θ scan probes only out-of-plane (lamellar) texture, these measurements are interpreted in terms of out-of-plane ordering and coherence and are not used to infer total crystallinity, unit-cell contraction, or the density of states. Additionally, an AFM surface scan was performed, as described in Section S2.3.2 of the Supplementary Materials. The AFM images show no clear differences in surface morphology among samples subjected to different annealing times.

#### 2.2.3. Optical Characterization

SE was employed to analyze the optical properties of the active layers of P3HT over a wavelength range of 300–900 nm. The ellipsometric parameters psi (ψ) and delta (δ), which describe the change in polarization state upon reflection, were measured and used to extract the complex dielectric function $\varepsilon_{r}=\varepsilon_{1}-i\varepsilon_{2}$, where $\varepsilon_{1}$, $\varepsilon_{2}$, the real and imaginary components, respectively, of the relative permittivity $\varepsilon_{r}$ of the films. The dielectric function was determined using the standard inversion $N=\sqrt{\varepsilon_{r}}=\sqrt{\varepsilon_{1}-i\varepsilon_{2}}$ [49,50,51]. The dielectric function was extracted with an isotropic effective model. A single angle of incidence does not provide enough independent information to determine the full uniaxial tensor, so we did not attempt to separate the in-plane and out-of-plane components. The same model and procedure were applied to every sample, so any systematic error from the isotropic approximation is common to the series and cancels in the relative comparison on which our conclusions rest. Each sample was also measured at several sites and gave reproducible spectra, confirming that the critical-point descriptors are stable features of the film. More details available in Section S2.2 of the Supplementary Materials. These optical, morphological, and electrical characterization methods provide informative data on electronic transitions, molecular ordering, and charge transport in the active layer as a function of annealing time.

## 3. Results and Discussion

Figure 2 shows the output characteristic *I–V* curves of the P3HT-based OTFT for different gate voltages (*V_G_*).

Figure 3 shows the normalized drain current (*I_D_*) results as extracted from the *I–V* measurement, alongside the normalized effective mobility results and morphological characteristics obtained from XRD analysis of the rr-P3HT-based OTFT active layer, as a function of annealing time. Figure 3a exhibits a monotonic enhancement in normalized drain current with increasing annealing time (0–30 min), extracted at a fixed gate voltage of $V_{G}=-30$ V. The drain current ($I_{D}$) values used to evaluate the electrical performance, were extracted from the saturation regime of the $I_{D}$–$V_{D}$ characteristics, where the drain current $I_{D}$ becomes independent of $V_{D}$ ($|V_{D}\left|\geq \right|\left(V_{G}-V_{T}\right)|$), as shown in Figure 2. Similar behavior is observed in Figure 3b for the effective mobility. All values are normalized to the as-cast (unannealed) sample to highlight the relative performance improvements due to thermal treatment. A monotonic increase is observed, with a particularly pronounced rise between 20 and 30 min, suggesting significant microstructural refinement in the active layer during prolonged annealing.

The effective mobility, $\mu_{eff}$, and threshold voltage, $V_{T}$, as a function of annealing time are summarized in Table 1. The extracted effective mobility exhibits an overall increase with annealing duration, rising from $5.8\times 10^{-4}$ cm^2^ V^−1^ s^−1^ in the as-cast device to $5.3\times 10^{-3}$ cm^2^ V^−1^ s^−1^ after 30 min of annealing. The increase is gradual up to 20 min, followed by a pronounced enhancement between 20 and 30 min. The effective mobility exhibits an overall increasing trend with annealing duration, although the increase is not strictly monotonic. This behavior suggests that the relationship between thermal annealing and charge transport is not straightforward. Although annealing can promote molecular reorganization and improve charge transport, the resulting electrical response may also depend on changes in energetic disorder, charge trapping, and contact-related effects. The pronounced mobility enhancement between 20 and 30 min may therefore reflect a change in the balance between these contributions. However, the available results do not establish a specific mechanism responsible for the intermediate decrease or subsequent increase in mobility. For comparison, the intrinsic low-field mobility ($\mu_{0}$) [47], exhibits a broadly similar trend, although a modest decrease is observed after 20 min of annealing. This difference between the two mobility extraction methods suggests that the measured transport properties may be influenced by factors beyond intrinsic channel mobility.

To investigate the structural origins of this current and mobility improvement, XRD measurements were conducted. As shown in Figure 3c, the diffraction peak near 2θ∼5.2°, corresponding to the (100) lamellar stacking plane of rr-P3HT [22,25,52], becomes more intense with increasing annealing time. A quantitative peak-fit analysis, including the peak position, full width at half maximum (FWHM), and integrated peak area for each annealing time, was performed to determine whether the peak exhibits a systematic narrowing with annealing, as summarized in Table 2.

The fitted widths, with an uncertainty of 0.01°, and the integrated area both increase from the as-cast state to 5 min of annealing. Between 5 and 10 min, the FWHM remains essentially unchanged, while the integrated area increases. Subsequently, between 10 and 20 min, both the FWHM and integrated area decrease, and both increase slightly again from 20 to 30 min. Notably, after 30 min of annealing, the maximum intensity, FWHM, and integrated area are all comparable to those observed after 10 min. The decrease in FWHM and integrated area between 10 and 20 min may be associated with enhanced out-of-plane lamellar ordering and coherence, which can contribute to more efficient charge-transport pathways [1,8,53,54,55,56]. However, this behavior does not directly correlate with the observed evolution of the drain current. The coherence length was estimated using the Scherrer equation [57,58], as summarized in Table 2. The coherence length obtained from the (100) reflection primarily represents structural coherence along the lamellar stacking direction [57]. The results indicate that, although annealing substantially changes the intensity of the (100) reflection, the coherent domain size remains approximately 10–12 nm, with an uncertainty of ∼1 nm. This suggests that the increase in crystalline ordering contributing to the (100) reflection does not necessarily correspond to a substantial increase in the coherent domain size. Moreover, charge transport in P3HT is strongly influenced by π-π stacking and interchain and interdomain connectivity, which are not fully captured by the coherence length obtained from the (100) reflection. Thus, the XRD results suggest that annealing modifies the degree and/or population of lamellar ordering, while the characteristic coherent domain size remains relatively unchanged. The apparent discrepancy between the structural parameters obtained from XRD and the electrical performance suggests that macroscopic crystallinity alone does not fully account for the enhanced charge transport. Localized morphological features or nanoscale ordering may not be adequately resolved by conventional XRD, which averages structural information over a relatively large sample area (∼10 mm^2^). In addition, several factors that are not directly captured by a symmetric XRD measurement could contribute to the observed behavior, including contact resistance, trap density, molecular orientation, optical anisotropy, and vertical structural heterogeneity between the film bulk and the channel interface. These factors are considered possible contributions rather than demonstrated mechanisms. The non-monotonic evolution of the diffraction intensity may also reflect competing nucleation and grain-growth processes or changes in the crystallite-size distribution.

Figure 4 presents the imaginary part of the relative permittivity, $\varepsilon_{2}$, as a function of photon energy for samples annealed from 0 to 30 min. Three distinct absorption peaks are observed at approximately 2.05 eV (605 nm), 2.24 eV (554 nm), and 2.38 eV (520 nm), corresponding to the 0–0, 0–1, and 0–2 optical transitions of the rr-P3HT, respectively. Notably, the 0–0 peak (∼2.05 eV), which appears as a weak “shoulder” in the unannealed sample, becomes significantly more pronounced with increased annealing time. This spectral evolution is indicative of improved lamellar stacking, consistent with increased molecular ordering [1,16,34,59], which modulates the optical transitions through changes in electronic coupling [60].

For comparison, simpler optical metrics derived from the same $\varepsilon_{2}$ data were also considered, including the Urbach energy of the absorption edge and the oscillator strength obtained from a single-Gaussian fit (Table S2 in Section S2.2 of the Supplementary Materials). The Urbach energy shows a modest reduction upon annealing, followed by an approximately constant value after 10 min. This behavior is similar to that observed for the XRD peak parameters and suggests that these relatively simple optical metrics are comparatively insensitive to the morphological changes occurring after the initial annealing stage. Taken together, the electrical, structural, and optical measurements demonstrate that annealing modifies the P3HT active layer and its charge-transport properties, although the evolution is not fully captured by conventional structural or optical descriptors. Overall, these findings emphasize the need for complementary characterization techniques to establish a more complete structure-performance relationship in P3HT-based OTFTs. In this context, XRD and SE should be regarded as complementary rather than competing probes, since they provide information on different structural and electronic characteristics and operate over different effective length scales. While XRD provides insight into out-of-plane crystalline ordering, SE reveals modifications in excitonic structure. These measurement techniques primarily provide qualitative insights and do not offer a complete quantitative framework for describing the underlying relationship between morphology and electrical performance. This limitation necessitates the implementation of a quantitative model to extract meaningful optical parameters that can be directly correlated with structural evolution and transport characteristics.

### 3.1. CP Model

We analyze the dielectric function extracted from SE data using the CP model, offering a more detailed and quantitative approach to probe subtle morphological changes through the optical transitions. Originally formulated by Cardona and co-workers [36,61], the CP model provides a theoretical framework to describe optical transitions near critical points, that is, energies where the JDOS exhibits abrupt changes. These transitions dominate the imaginary part of the relative permittivity, $\varepsilon_{2}$, and reflect interband excitations in materials, directly influenced by the material’s electronic structure, morphology, and degree of order. In conjugated polymers like P3HT, these transitions stem from π-$\pi^{*}$ excitations and excitonic effects, which are highly sensitive to molecular ordering and chain packing [49,62].

The CP model expresses the dielectric response as a sum of individual contributions from various dimensionalities of critical points (0D, 1D, 2D, 3D), formulated as [36,49,60,62,63]:

(1) $$\varepsilon \left(E\right)=C-\sum_{j}^{\#CP}P_{j}\left(E\right)$$

(2) $$P_{j}\left(E\right)=\left\{\begin{array}{cc} e^{i\phi_{j}}\frac{A_{j}}{{\left(E-E_{0,j}+i\Gamma_{j}\right)}^{\frac{2-n_{j}}{2}}} & n_{j}\neq 2\\ e^{i\phi_{j}}\cdot \ln\left(\frac{A_{j}}{E-E_{0,j}+i\Gamma_{j}}\right) & n_{j}=2 \end{array}\right.,\;$$

where ε(E) is the dielectric constant as a function of energy (E), and the dimensionality of the transition (0–3D), which determines the energy dependence of the singularity, is given by $n_{j}$. Each critical point contribution $P_{j}\left(E\right)$ is characterized by $E_{0,j}$, $A_{j}$, $\phi_{j}$, and $\Gamma_{j}$, which are the transition energy, amplitude, phase, and broadening factor, respectively, for the $j^{th}$ critical point, and the constant *C* is a real, energy-independent term. The dimensionality parameter ($n_{j}$) determines the functional form of the spectral singularity and is defined as a discrete integer corresponding to the critical-point model. Accordingly, the dimensionality is treated as a discrete model-selection variable, with candidate integer values evaluated through nonlinear fitting of the corresponding CP expressions. The transition energy $E_{0,j}$ indicating the onset of interband transitions (eV); the amplitude $A_{j}$ related to the oscillator strength of the transition, carries units consistent with Equation (2) (ε being dimensionless); phase $\phi_{j}$ is a real angle (radians), accounting for asymmetric line shapes due to interactions and inhomogeneous broadening, bounded within 0 to 2π; broadening $\Gamma_{j}$ is the broadening (damping) parameter of the line shape, equal to half the FWHM only for the zero-phase 0D Lorentzian case, with no universal FWHM conversion for other phases or dimensionalities, linked to structural disorder and exciton localization [49,63,64,65]. Together, these parameters define the shape and physical origin of absorption peaks in $\varepsilon_{2}\left(E\right)$, offering a quantitative link between morphology and optoelectronic properties.

#### Fitting the CP Model to the SE Data

Fitting the CP model to experimental data is inherently nonlinear due to its complex functional form and mathematical structure, including complex exponents that do not vary linearly with respect to the fitting variables. Each critical point is described by a complex function that includes nonlinear dependencies on several interrelated parameters, such as the transition energy ($E_{0,j}$), amplitude ($A_{j}$), broadening ($\Gamma_{j}$), dimensionality ($n_{j}$), and phase ($\phi_{j}$). This nonlinearity presents two significant implications for the fitting process: (i) Multiple local minima: The error surface defined by the difference between the experimental and modeled dielectric functions is highly non-convex, meaning that traditional gradient-based optimization methods can easily become trapped in local minima. This makes it essential to use global optimization strategies, such as multi-start algorithms, which run the fitting process multiple times with varied initial guesses to improve the likelihood of finding the global minimum. (ii) Parameter coupling: Several CP parameters do not independently vary in fit. For example, changes in $\Gamma_{j}$ can affect both the amplitude and phase of the feature, and vice versa. This coupling increases the complexity of the fitting landscape. It necessitates robust numerical optimization techniques, such as nonlinear least squares solvers (e.g., Levenberg–Marquardt), which are well-suited to handling interdependent parameters. Additionally, the combinatorial complexity of the model increases the nonlinear nature of the fitting. In this study, we implemented the CP model in MATLAB R2023b (academic license) using a multi-start nonlinear optimization algorithm. This approach was chosen to explore the parameter space effectively and avoid convergence to suboptimal solutions. More details available in Section S3 of the Supplementary Materials. The CP model was fitted to $\varepsilon_{2}$ spectra for all annealing times (0–30 min). As shown in Figure 5, the model reproduces an agreement between experimental $\varepsilon_{2}$ spectra and model outputs, with the mean-squared error (MSE) reported in Table S3 in the Supplementary Materials (the MSE is a goodness-of-fit measure and is not equivalent to a percentage error). While prior studies have used up to five CP transitions to model polymer thin films [5], our analysis consistently identified three dominant transitions located at ∼2.03 eV (CP1), ∼2.20 eV (CP2), and 2.36 eV (CP3). These correspond closely with the optical 0–0, 0–1, and 0–2 transitions of P3HT, respectively [16,21,22,25,33,34,35].

Figure 6 reveals how individual CP transition peaks evolve with annealing time. Notably, CP1 (∼2.03 eV), associated with the 0–0 transition, shows a marked increase in amplitude and sharpness with annealing time. These attributes signify improved molecular order and π-π stacking interactions [1,16]. CP2 and CP3 display more moderate evolution, indicating that CP1 is the most sensitive probe of structural refinement in P3HT.

To benchmark the CP analysis, we additionally considered Spano’s H-/J-aggregate model, which describes the evolution of excitonic coupling in conjugated polymers via optical transition peak ratios [32,60,66]. Analogous to the A_0−0_/A_0−1_ ratio in Spano’s model, our extracted CP1/CP2 ratio follows a qualitatively similar trend, although the two quantities are not identical: the CP amplitudes derive from fitted critical-point line shapes rather than resolved vibronic 0–0 and 0–1 intensities, so we do not equate CP1/CP2 with Spano’s A_0−0_/A_0−1_, nor do we extract an exciton bandwidth W from it. Figure 7 further emphasizes the significance of CP1. Figure 7a shows the CP1/CP2 peak area ratio, which rises sharply between 10 and 20 min of annealing, from 0.29 to 1.26, signaling a transition in the film’s microstructure. This transition is mirrored by a steady increase in normalized CP1 intensity (Figure 7b), reinforcing its role as an optical indicator of molecular ordering and providing quantitative support for the increased molecular order and crystallinity that occur with annealing. In parallel, the broadening parameter $\Gamma_{j}$, shown in Figure 8, decreases steadily with annealing time for CP1 and CP2, whereas CP3 evolves non-monotonically (broadening increases slightly between 20 and 30 min). The reduction in $\Gamma_{j}$ for CP1 indicates a decreased energetic disorder, corresponding to enhanced exciton delocalization and improved electronic coherence, which are expected to influence charge transport. We note that Γ contains both homogeneous (lifetime/exciton–phonon) and inhomogeneous (static disorder) contributions, which the present data cannot separate. Importantly, these optical changes are consistent with dimensionality transitions captured through the CP model. The peak’s dimensionality is a unique property associated with the CP model and is not shared by any other model. Table 3 summarizes the evolution of dimensionality as a function of annealing time. The evolution from 0D to 1D in CP1 reflects a transition from localized electronic states in disordered regions to more extended (delocalized) electronic states, consistent with reduced energetic disorder. We emphasize that this dimensionality is a parameter describing the energy dependence of the JDOS near the critical point and does not, by itself, establish geometrically interconnected crystalline domains or transport dimensionality. This subtle change in dimensionality after 20 min of annealing precedes the previously observed increase in the current after 30 min of annealing.

Table 4 provides a comprehensive summary of key optical and electrical parameters extracted as a function of annealing time, highlighting correlations between molecular ordering and charge-transport performance in P3HT-based OTFTs. The dimensionality of CP1 transitions from 0D to 1D after 20 min of annealing, which coincides with a significant increase in the CP1/CP2 peak area ratio, from 0.29 at 10 min to 1.26 at 20 min. Furthermore, the broadening parameter $\Gamma_{CP1}$ decreases steadily from 89.7 to 67.4 meV, with the most significant change occurring between 10 and 20 min (from 86.6 meV to 70.2 meV), suggesting a narrowing of the density of states and improved electronic coherence. Although an immediate change in electrical performance that directly mirrors the variations in the model parameters is not observed, the most significant increase in drain current occurs between 20 and 30 min of annealing, indicating a consistent trend. Changes in contact resistance, trap density, or other device-level factors during annealing may contribute to this difference, since the optical descriptors primarily probe the active-layer properties. We therefore describe this relationship as a correlation on a single benchmark series rather than a validated predictive relationship: with five annealing-time points that share annealing time as the common ordering variable, a predictive model cannot be established. Nevertheless, the Pearson correlation analysis between the CP descriptors and electrical metrics provides quantitative support for the observed trends, with correlation coefficients of approximately R=0.85 (see Table S4 in the Supplementary Materials). The consistency observed across multiple CP descriptors further indicates that the CP parameters capture systematic changes in the optical response associated with annealing-induced evolution of the P3HT film. Importantly, the CP analysis provides information that is not readily accessible from XRD alone. In particular, the evolution of the CP parameters associated with the 0–0 (CP1) transition provides sensitive quantitative descriptors of changes in the electronic structure and molecular ordering. These parameters can therefore serve as useful indicators for identifying processing conditions under which substantial improvements in electrical performance may be expected. While further measurements across independent processing series and device structures are required to establish predictive capability, the present results demonstrate the potential of the CP model as a quantitative framework for linking optical and morphological evolution to charge transport and for guiding the optimization of P3HT-based OTFT processing conditions.

Using the CP model on the P3HT-based OTFTs enables a quantitative framework for correlating optical transitions with morphological evolution, offering a more profound and rigorous interpretation of the observed results. The consistent trends observed in dimensionality parameters, peak area ratios, and line-broadening factors Γ reveal a coherent picture of the film’s structural ordering and electronic landscape evolution with increasing annealing time. Among all CPs, CP1 consistently emerges as the most sensitive optical indicator of molecular ordering, as evidenced by its increasing amplitude, narrowing linewidth, and shift in dimensionality, all of which correlate with electrical performance in OTFT devices. The evolution of CP1 from 0D to 1D character reflects increased electronic delocalization and reduced disorder, correlating with the improvements in molecular ordering and charge transport inferred from the other measurements. Moreover, the increasing CP1/CP2 peak area ratio follows a trend qualitatively similar to that expected from Spano’s excitonic coupling picture, without asserting quantitative equivalence. These findings support the connection between improved molecular ordering and enhanced charge transport, despite potential discrepancies due to additional factors not accounted for in the model, such as trap density, contact resistance, and other extrinsic effects that influence charge transport. By directly relating to the joint density of states, the CP model provides a physically grounded framework to translate subtle optical features into quantitative descriptors of morphology and electronic structure. The model provides a physically grounded description of how processing affects optoelectronic properties by fitting the complex dielectric function with a set of physically meaningful parameters: transition energy, amplitude, and broadening. Importantly, these optical parameters support the structural improvements observed by XRD and SE and offer a reliable, non-destructive tool for screening the relative evolution of device-relevant film order using optical measurements. These findings reinforce the utility of the CP model as a diagnostic tool for tailoring processing conditions in organic electronic devices.

## 4. Conclusions

In this study, poly(3-hexylthiophene) (P3HT)-based organic thin-film transistors (OTFTs) were fabricated by spin coating and subjected to controlled thermal annealing for different durations at 140 °C. Electrical characterization revealed a non-monotonic evolution of the drain current and extracted mobility as a function of annealing time, with the most pronounced improvement occurring between 20 and 30 min. X-ray diffraction (XRD) measurements indicated changes in out-of-plane lamellar ordering, while atomic force microscopy (AFM) measurements showed no pronounced differences in the surface morphology between the different annealing conditions. These results did not fully align with electrical performance, highlighting limitations in conventional structural analysis. To address this, we employed spectroscopic ellipsometry (SE) in conjunction with the critical point (CP) model to extract quantitative optical parameters related to the active layer’s morphology. Importantly, the CP-derived parameters complement the structural information from XRD and the optical response from SE, providing a more detailed picture of the processing-dependent evolution of the active layer. The non-destructive nature of SE further makes this approach attractive for screening relative changes in device-relevant film properties without requiring device fabrication at every stage. Although validation across independent processing conditions and device structures is required before predictive capability can be established, the present results demonstrate that CP analysis can serve as a sensitive diagnostic framework for evaluating processing-induced changes in organic semiconductor films and for guiding the optimization of organic electronic devices. CP analysis resolved systematic changes in the dielectric response through physically meaningful parameters, including transition dimensionality, amplitude, and broadening. In particular, the CP1 transition at approximately 2.03 eV, corresponding to the 0–0 optical transition, exhibited a transition from 0D to 1D character with annealing, accompanied by a reduction in its linewidth and an increase in its relative contribution with respect to CP2. These changes are consistent with increased electronic delocalization and reduced disorder associated with improved molecular ordering. The increase in the CP1/CP2 peak-area ratio also follows a trend qualitatively consistent with the evolution expected from Spano’s excitonic-coupling picture, although quantitative equivalence between the CP parameters and excitonic coupling parameters is not assumed. The combined evolution of these parameters supports a relationship among the optical response, molecular ordering, and charge transport, while recognizing that electrical performance is also affected by factors not explicitly captured by the optical model, including trap density, contact resistance, *etc*. Because the CP formalism describes the dielectric response in terms of transition energy, amplitude, broadening, and dimensionality, it enables subtle changes in the electronic structure to be distinguished more effectively than simpler spectral metrics, providing a set of physically meaningful descriptors with which systematic changes induced by processing can be quantified. This study was intentionally designed as a single-temperature benchmark in which annealing time was isolated as the principal processing variable. The limited number of annealing conditions is sufficient to identify systematic trends but insufficient to enable statistical generalization or a rigorous assessment of process-to-process variability. Future studies should therefore include a larger number of samples and independent device replicates, as well as multiple annealing temperatures and processing conditions. Direct structural probes such as AFM, GIWAXS, and TEM would also provide valuable independent tests of the structural interpretation developed from the optical measurements. In addition, dedicated contact-resistance test structures could provide a more direct assessment of the contribution of contact effects to the observed electrical evolution. In our view, CP/SE analysis complements rather than replaces AFM and XRD: AFM provides surface topography, and XRD provides out-of-plane crystalline ordering and lattice spacing, while CP/SE adds a rapid, non-destructive optical read-out sensitive to subtle changes in electronic order and disorder. Its role is therefore early, fast screening of relative electronic-order evolution during process development, narrowing a processing window before slower structural characterization, rather than a substitute for it. Overall, this work demonstrates that the CP model, applied to SE data, is a non-destructive tool for probing microstructural evolution in organic semiconductors. It provides a rapid, non-destructive optical read-out that correlates with OTFT performance and can complement electrical testing during process development; establishing genuine predictive capability will require validation across more samples, repeats, and processing conditions. These insights deepen our fundamental understanding of the structure–property relationships in P3HT-based systems and provide a practical route toward improved process characterization for organic electronics.

## Figures and Tables

**Figure 1:**
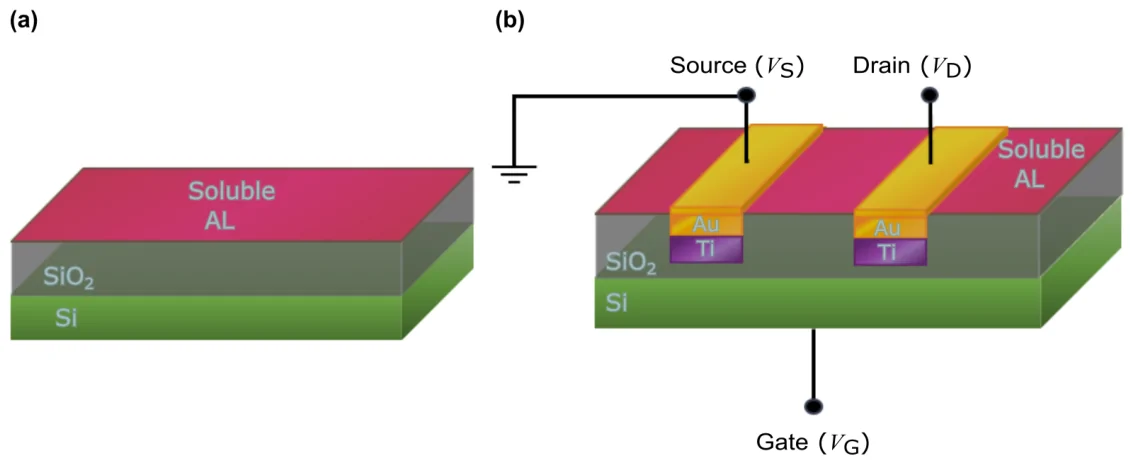
Schematic of the organic thin-film transistor (OTFT) fabrication process. (**a**) A SiO_2_/Si substrate coated with a P3HT organic polymer active layer (AL). (**b**) Complete OTFT structure: silicon substrate (gate), SiO_2_ dielectric isolating layer, gold (Au) source (S) and drain (D) electrodes with titanium (Ti) adhesion layer, and the spin-coated organic semiconducting layer (P3HT).

**Figure 2:**
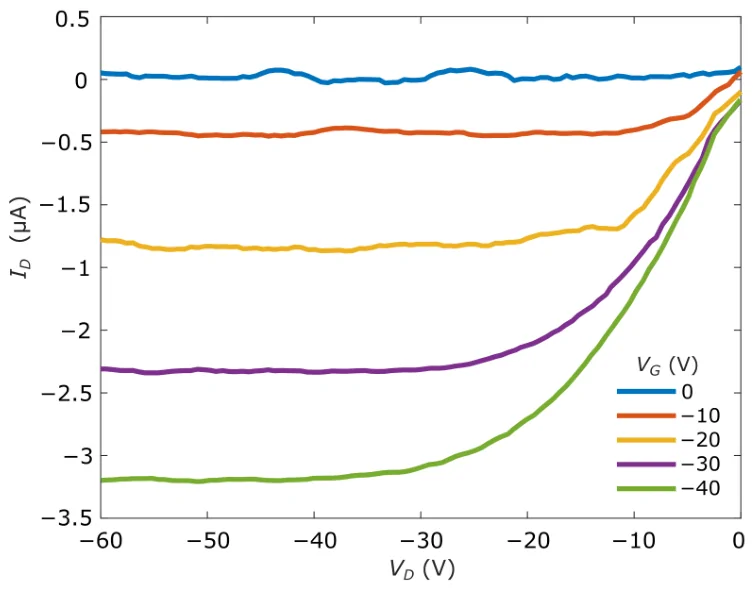
Electrical performance of the P3HT-based OTFT output characteristics $I_{D}$–$V_{D}$ curves at a different gate voltages from 0 to −40 V, after 10 min of annealing.

**Figure 3:**
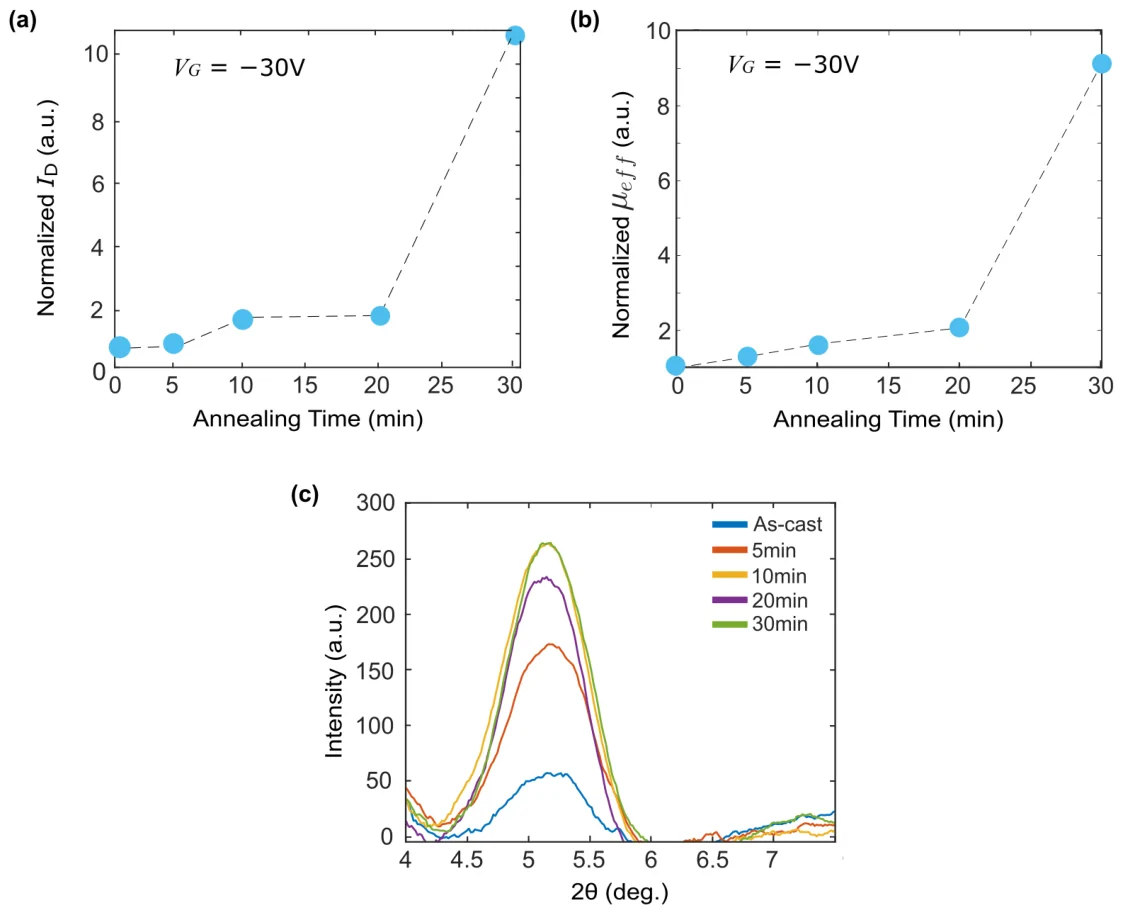
Electrical and morphological characterization of rr-P3HT-based organic thin-film transistors’ active layer under different annealing times: (**a**) Normalized drain current (*I_D_*), extracted from the *I–V* curves at a constant gate voltage of $V_{G}=-30$ V, as a function of annealing time. (**b**) Normalized effective mobility extracted by the Y-function method at a different annealing time. (**c**) X-ray diffraction (XRD) maximum intensity at the P3HT characterized angle of 2θ∼5.2° as it was measured for each film at a different annealing time.

**Figure 4:**
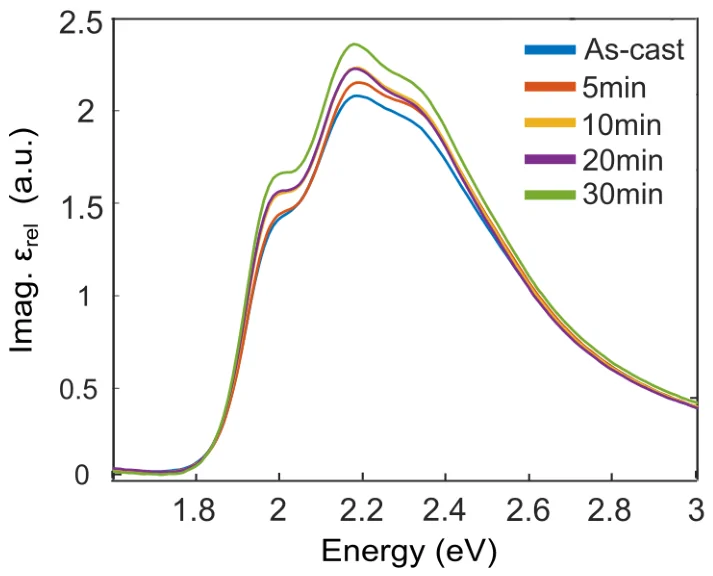
Optical characterization of the P3HT-based organic thin-film transistors’ active layer as a function of annealing time. The imaginary component of the relative permittivity ($\varepsilon_{2}$), extracted from spectroscopic ellipsometry measurements, is shown as a function of photon energy (corresponding to the 300–900 nm wavelength range) for samples annealed for durations ranging from 0 to 30 min.

**Figure 5:**
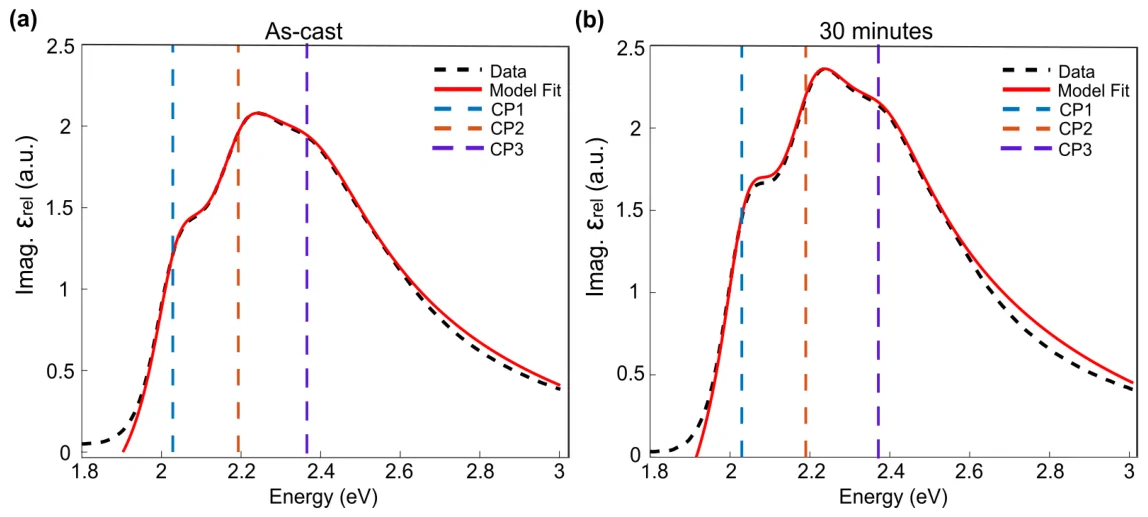
Critical point model fitting to SE data for two different annealing durations: (**a**) As-cast. (**b**) 30 min. The black dashed line shows the experimental imaginary part of the relative permittivity $\varepsilon_{2}$, with CP transitions indicated by vertical dashed lines.

**Figure 6:**
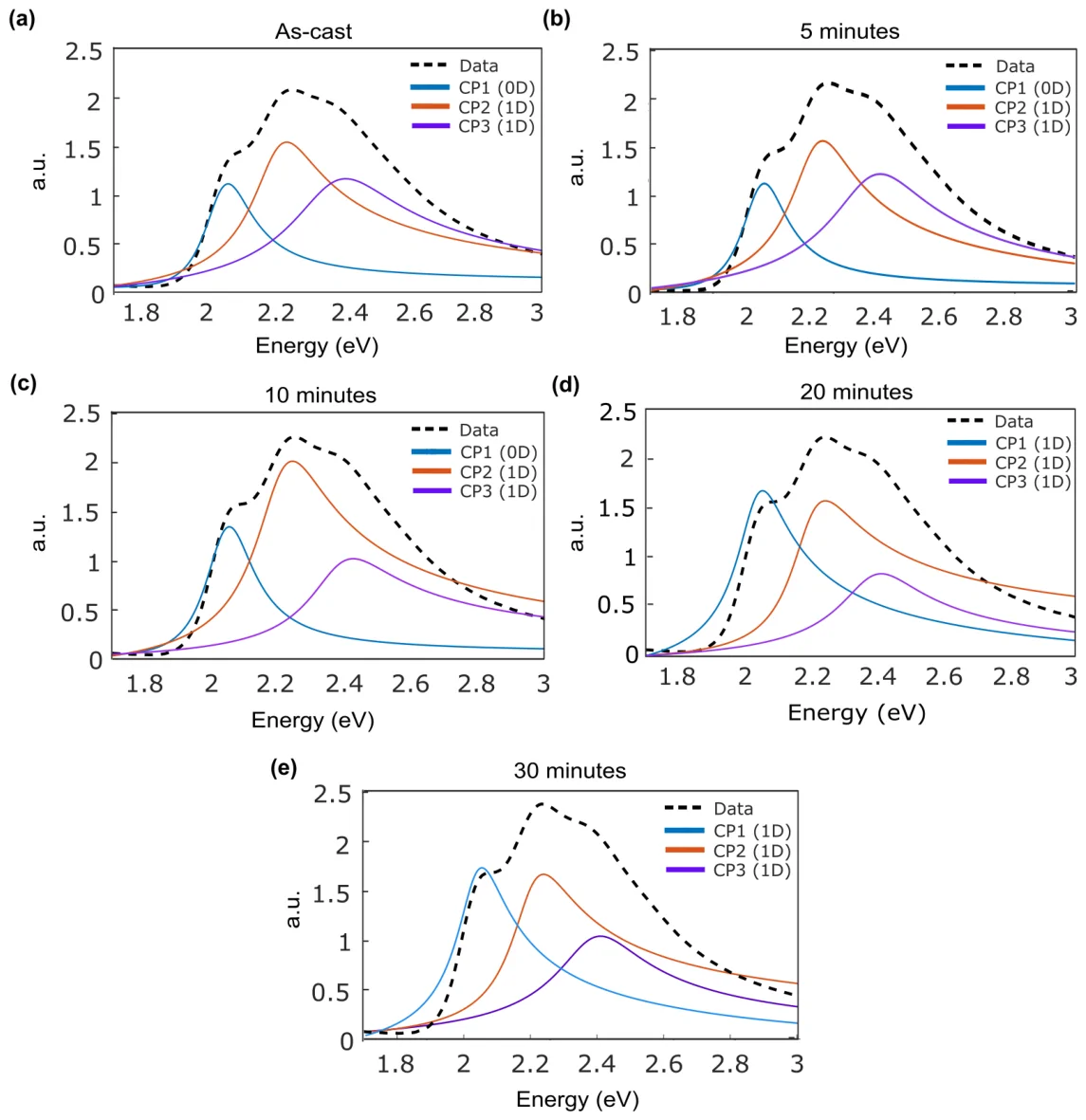
Individual critical point (CP) peak fittings for the imaginary part of the relative permittivity as a function of photon energy across annealing times: (**a**) As-cast, (**b**) 5 min, (**c**) 10 min, (**d**) 20 min, and (**e**) 30 min.

**Figure 7:**
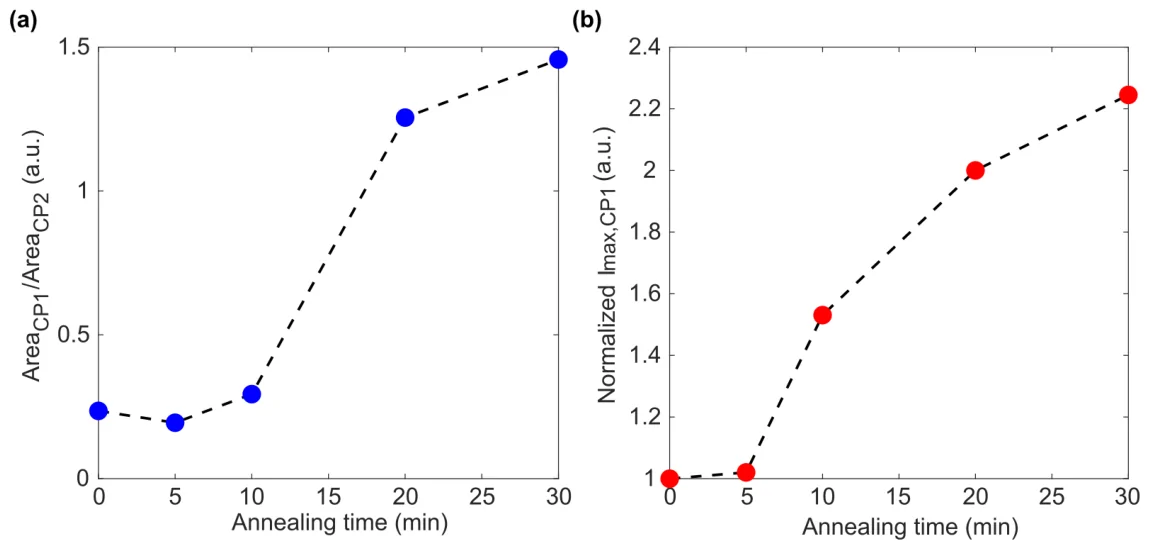
Evolution of critical point features with annealing. (**a**) Ratio of CP1 (2.03 eV) to CP2 (2.20 eV) peak areas. (**b**) Normalized maximum intensity of CP1 as a function of annealing time. Values are normalized to the as-cast sample.

**Figure 8:**
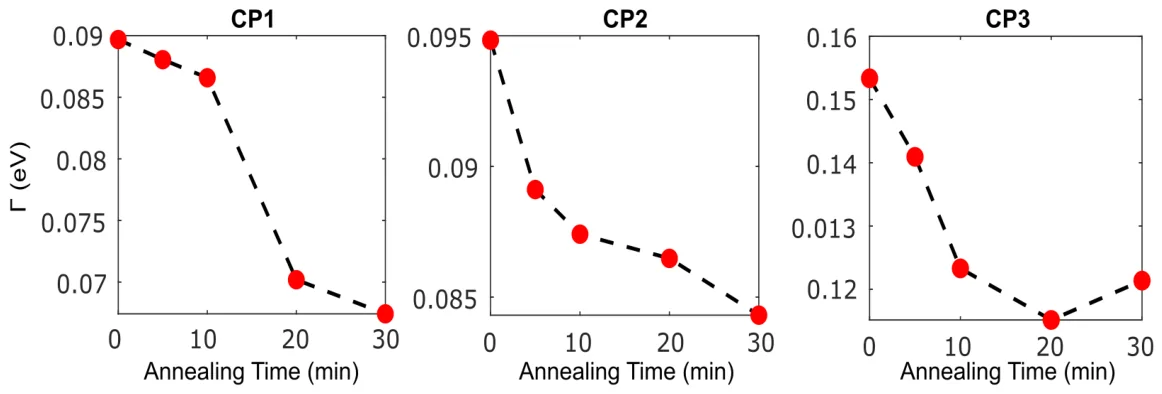
Variation of Gamma (Γ) parameter for each critical point (CP) as a function of annealing time.

**Table 1:** Extracted effective mobility ($\mu_{eff}$), and threshold voltage ($V_{T}$) as a function of annealing time.

Annealing	$\mu_{\mathrm{eff}}$	Threshold
Time (min)	(cm^2^ V^−1^ s^−1^)	Voltage (V)
0	$5.8\times 10^{-4}$	16.0
5	$7.5\times 10^{-4}$	7.9
10	$9.5\times 10^{-4}$	5.8
20	$1.2\times 10^{-3}$	9.1
30	$5.3\times 10^{-3}$	9.7

**Table 2:** A quantitative X-ray diffraction (XRD) peak-fit and spectroscopic ellipsometry (SE) analysis results at various annealing times.

Annealing	Max	FWHM	Integrated	$L_{c}$
Time (min)	Intensity (a.u.)	(deg)	Area (a.u.)	(nm)
0	57	0.68	40	11.7
5	173	0.77	136	10.3
10	264	0.78	205	10.2
20	234	0.72	170	11.1
30	264	0.76	201	11.5

**Table 3:** Dimensionality parameter for each critical point (CP) obtained from model fitting at various annealing times. The bold numbers indicate a change in dimensionality.

Annealing Time (min)		Dimension (n)	
	CP1	CP2	CP3
	∼2.03 (eV)	∼2.2 (eV)	∼2.36 (eV)
0	0	1	1
5	0	1	1
10	0	1	1
20	1	1	1
30	1	1	1

**Table 4:** Comparison of key optical and electrical parameters with annealing time: CP1 dimensionality, CP1/CP2 area ratio, CP1 broadening parameter ($\Gamma_{\mathrm{CP}1}$), transistor’s drain current at saturation, and extracted effective mobility ($\mu_{eff}$).

Annealing	CP1	CP1/CP2	$\Gamma_{\mathrm{CP}1}$	Drain Current	$\mu_{\mathrm{eff}}$
Time (min)	Dimensionality	Area Ratio	(meV)	$\left|I_{D,\mathrm{sat}}\right|$ (μA)	(cm^2^ V^−1^ s^−1^)
0	0D	0.24	89.7	1.2	$5.8\cdot 10^{-4}$
5	0D	0.20	88.1	1.3	$7.5\cdot 10^{-4}$
10	0D	0.29	86.6	2.3	$9.5\cdot 10^{-4}$
20	1D	1.26	70.2	2.4	$1.2\cdot 10^{-3}$
30	1D	1.46	67.4	12.4	$5.3\cdot 10^{-3}$

## Data Availability

The data supporting the findings of this study (including MATLAB (R2023b) analysis scripts, raw SE spectra (Excel 2016), etc.) are available from the corresponding author upon reasonable request.

## Supplementary Materials

The following supporting information can be downloaded at: https://www.mdpi.com/article/10.3390/polym18212673/s1. References [67,68] are cited in the supplementary materials.
