# Supplementary material for: From Optical Transitions Through Morphology Changes to Charge Transport: A P3HT Benchmark Study Using the Critical Point Model
Source: Polymers (Basel). 2026 Oct 28;18(21):2673. doi: 10.3390/polym18212673 (PMC13765619; doi:10.3390/polym18212673)
Supplement: Supplementary file 1 [file polymers-18-02673-s001.zip › polymers-4559179-supplementary.pdf]

Supplementary Information (SI) for Journal of Polymers.

Supplementary Materials

From Optical Transitions through Morphology Changes to Charge Transport: A P3HT Benchmark Study Using the Critical Point Model

Tal Elbaz*[1], P.R. Chithira[1,3], and Rafi Shikler[1,2]

[1]School of Electrical & Computer Engineering, Ben-Gurion University of the Negev, Beer-Sheva ,Israel

[2]Ilse Katz Institute for Nanoscale Science and Technology, Ben-Gurion University of the Negev, Beer-Sheva, Israel.

[3]Department of Physics, Presidency University, Bengaluru 560064, India.

*E-mail: talelb@post.bgu.ac.il

1. OTFT Fabrication

Organic thin-film transistors (OTFTs) were fabricated on p-doped silicon substrates (thickness: 525 µm, resistivity: 0.001-0.005 $\Omega \cdot cm$) with a thermally grown silicon dioxide (SiO_2) insulating layer (thickness: 90 nm). Prior to device fabrication, the substrates were sequentially cleaned by sonication in acetone, methanol, and isopropanol, followed by an oxygen plasma treatment to remove residual organic contaminants and improve surface wettability.

Gold (Au) source and drain electrodes (60 nm thick) were deposited via thermal evaporation (rate of $0.1\ \frac{nm}{s}$ for the first 10 nm, then $0.3\ \frac{nm}{s}$) under a vacuum of 2.8×10^{-6} torr, forming a spiral-patterned configuration. To enhance adhesion between the electrodes and the SiO_2 surface, a thin titanium (Ti) interlayer (5 nm thick) was deposited prior to the Au evaporation with evaporation rate of $0.1\ \frac{nm}{s}$. The channel length is $L = 10$ µm, and channel width is $W = 1$ mm

The semiconducting active layer (AL) solution was prepared from rr-P3HT, purchased from Ossila, (regioregular poly(3-hexylthiophene)) with molecular weight of M_w=60K and regioregularity of 97.6%. The solution concentration was 5 mg per 1 mL of chloroform, stirred at 40 °C for 2 hr to ensure complete dissolution and homogenization. The rr-P3HT film was then deposited onto the Au-patterned substrates by spin coating at 2,500 rpm for 60 s. Post-deposition, the films were thermally annealed on a pre-heated hot plate at 140 °C [1-3] inside a nitrogen-filled glovebox for various durations (5, 10, 20, and 30 min) to promote molecular reorganization and crystallinity. The blanket films used for XRD and SE and the OTFT devices were prepared from the same deposition batch and annealed together, ensuring batch-to-batch consistency. All samples were annealed separately.

2. OTFT Characterization

2.1 Electrical Performance

Electrical measurements were conducted using a Keithley 2636B source measurement unit. The gate voltage (V_G) was fixed at 0, -10, -20, -30, and −40 V, while the drain voltage (V_D) was swept from 0 V to −60 V to obtain the transistor characteristics $I_D - V_D$ curves. All data analysis and parameter extraction were performed using MATLAB (academic license).

a)

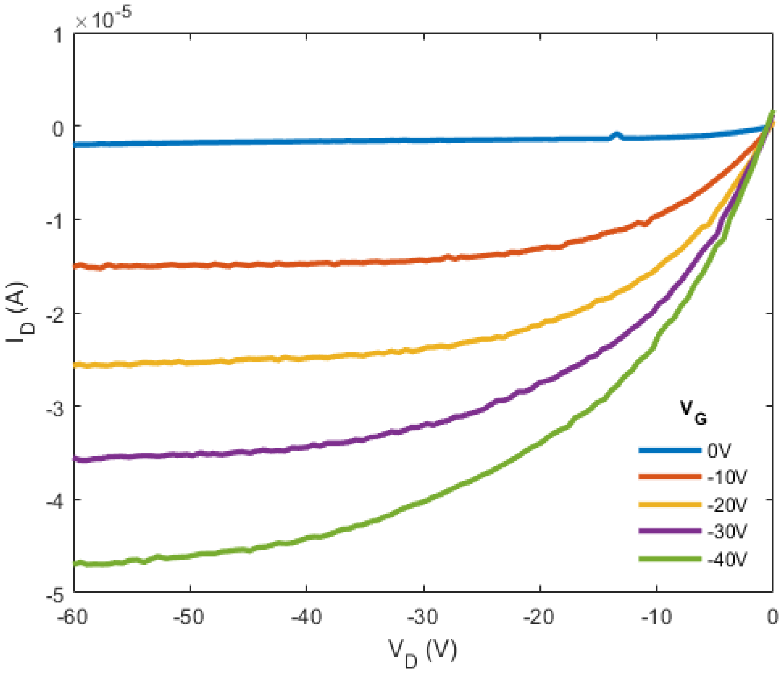

Figure S1. Measured *I-V* curves of P3HT-based OTFT after 10 minutes of annealing for another (2nd) experimental set.

The intrinsic mobility was extracted using the Y-function method [4-6], as shown in Table S1.

Table S1. Intrinsic mobility extraction using the Y-function method for different annealing time.

Annealing Time (min)	Y-function Mobility (cm^2/V s)	R^2
0	$2.4 \cdot 10^{-4}$	0.98
5	$6.6 \cdot 10^{-4}$	0.98
10	$1.6 \cdot 10^{-3}$	0.99
20	$4.1 \cdot 10^{-4}$	0.99
30	$7.1 \cdot 10^{-3}$	0.98

2.2 Optical Characterization

Optical characterization was performed using a J.A. Woollam Alpha-SE Ellipsometer at an incidence angle of 70°. Measurements were performed at different sites on the substrate to verify reproducibility. A three-layer optical model (Si / SiO_2 / P3HT) was employed, where we used a Cauchy model and a B-spline model for the fitting. The dielectric function ($\varepsilon = \varepsilon_1 - i\varepsilon_2$) of the P3HT active layer was extracted using MATLAB by fitting the experimental ellipsometric parameters ψ and Δ, based on the fundamental ellipsometric relation[7, 9]:

$$\rho \equiv tan\,\psi\, exp(i\Delta) \equiv \frac{r_p}{r_s} \;\;,$$

where ψ is the amplitude ratio, characterized by the refractive index n, and Δ is the phase difference between p- and s- polarizations representing light absorption, also expressed as:

$$tan\,\psi = \frac{|rp|}{|rs|} \;\;, \qquad \Delta = \delta_{rp} - \delta_{rs} \;\;.$$

Where r_p and r_s are the Fresnel reflection coefficients for p- and s-polarized light, respectively. The amplitude ratio and phase difference are defined as [7-9]:

The reflection coefficients were calculated using the Fresnel equations for each interface:

$$rjk,p = \frac{Nk\,cos\,\theta j - Nj\,cos\,\theta k}{Nk\,cos\,\theta j + Nj\,cos\,\theta k} \quad , \quad rjk,s = \frac{Nj\,cos\,\theta j - Nk\,cos\,\theta k}{Nj\,cos\,\theta j + Nk\,cos\,\theta k} \;,$$

where j, k denote the layer indices such that *j = 0, 1, 2, 3* and *k = j + 1,…,3* , with the complex refractive index convention $N \equiv n - ik$, and θj is the propagation angle in each layer. The dielectric function was determined using the standard inversion relation:

$$N \equiv \sqrt{\varepsilon_r} = \sqrt{\varepsilon_1 - i\varepsilon_2} \;\;.$$

As presented in Figure S2, for example.

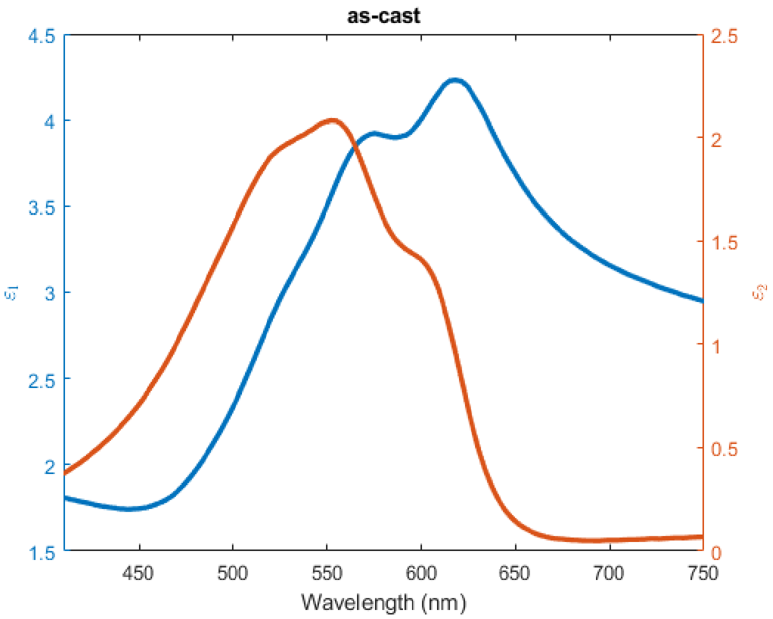

Figure S2. The real (blue) and imaginary (orange) parts of the dielectric function of the as-cast sample, as it was calculated out of the ellipsometry data.

Annealing produces a modest reduction in the energetic disorder of P3HT, followed by an approximately constant Urbach energy after 10 min. The most significant change appears to occur between 0 and 10 min.

Table S2. Urbach Energy of P3HT films at different annealing times. R^2 represents the residuals.

Annealing time (min)	E_U (meV)	R^2
0	57.61	0.999
5	55.68	0.999
10	54.67	0.998
20	54.99	0.998
30	54.54	0.998

2.3 Morphological Characterization

2.3.1 XRD scan

The XRD measurements were taken using a Panalytical Empyrean multi-purpose diffractometer using Cu Kα radiation (λ = 1.540 Å). XRD patterns were recorded in a $\theta/2\theta$ scan geometry over a 2θ range of 4° to 40°, step size of 0.01°, and a no-coat SiO_2/Si sample was measured as well for low background. The mean coherence length (Scherrer equation) was found to be $L_c = 11 \pm 1\ nm$ (the Scherrer constant was taken as K = 0.9).

One can see in Figure S3, that although changes in crystallinity were observed through CP model' analysis, relatively weak changes in the signal appear, owing to the limitations of the tool, i.e., noise at small angles or insensitivity.

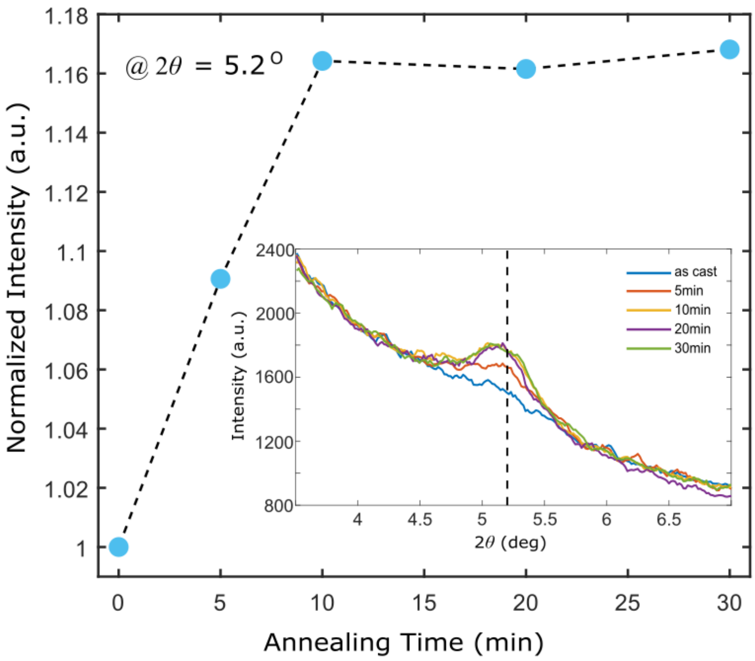

Figure S3. Normalized maximum intensity values (at $2\theta \sim 5.2°$) of the XRD measurement of P3HT films at different annealing times. The values are normalized to the as-cast sample. Inset **-** XRD measurement results for different annealing times of P3HT active layer.

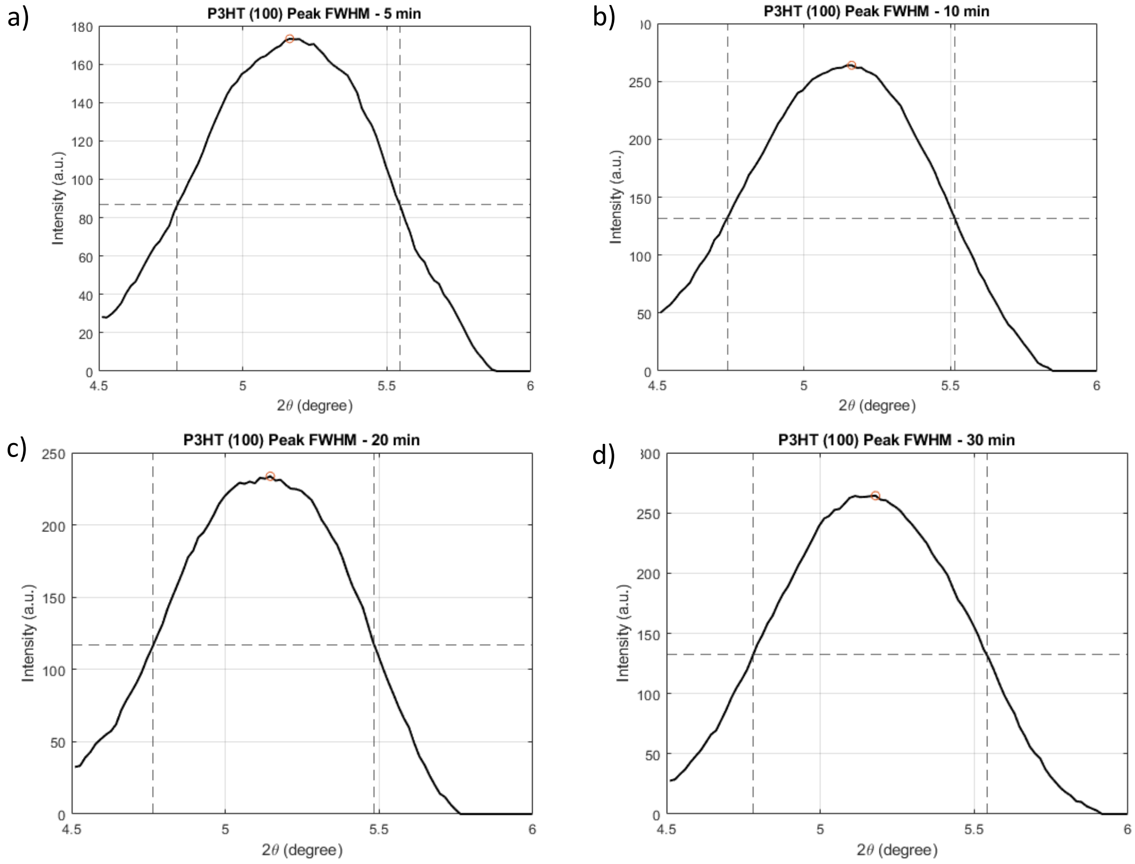

Figure S4. XRD intensity at $2\theta \sim 5.2°$ of P3HT films at different annealing times: a) 5 min, b) 10 min, c) 20 min, and d) 30 min. The FWHM is marked by dashed black lines.

2.3.2 AFM scan

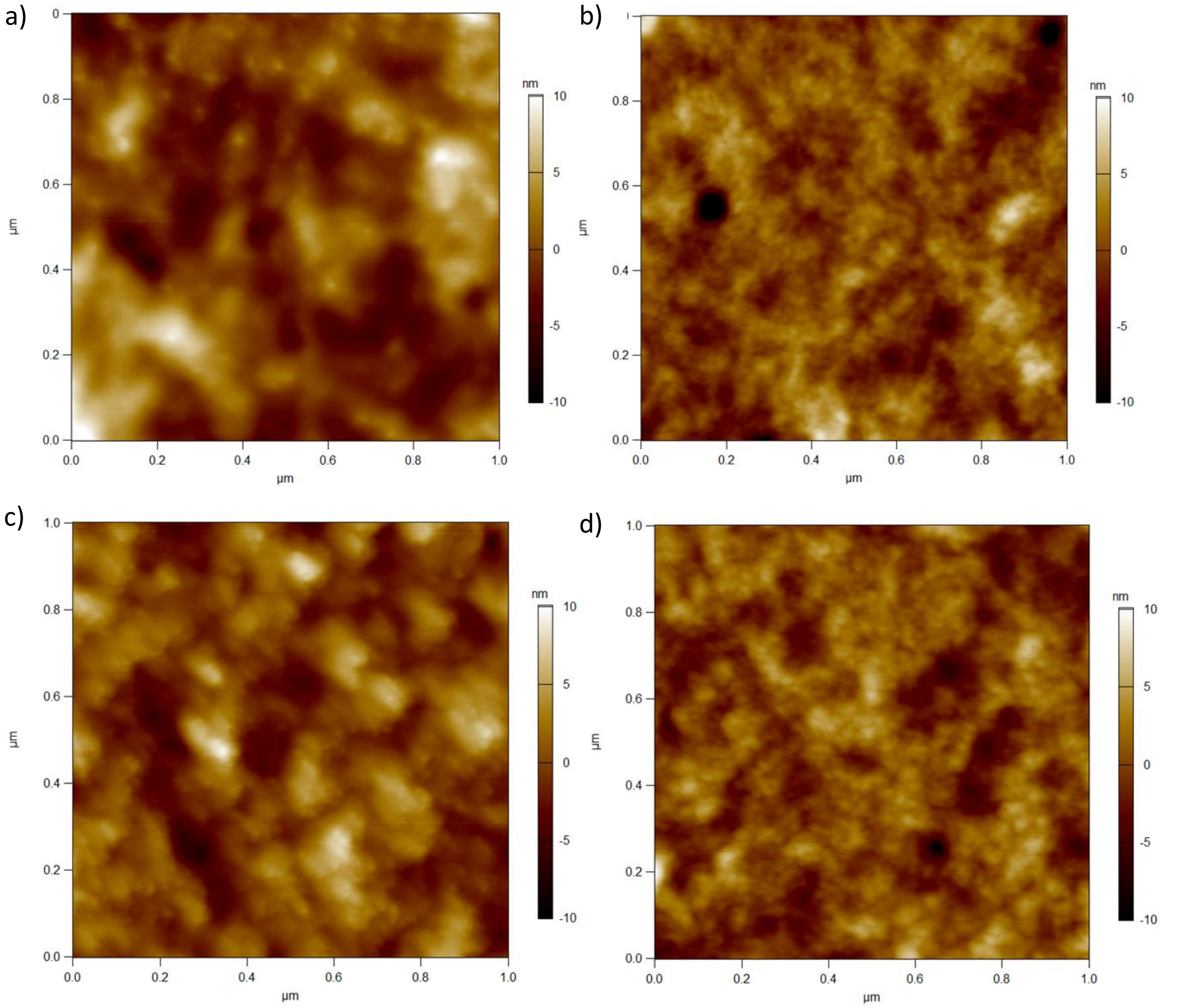

Figure S5. AFM scan of P3HT films at different annealing times: a) as-cast, b) 10 min, c) 20 min, and d) 30 min.

3. CP Model Construction

The Critical Point (CP) model analysis was conducted on the complex dielectric function ε(E) obtained from the ellipsometric model described in Section S1. The dielectric function, derived from spectroscopic ellipsometry (SE) measurements and modeled using MATLAB, served as the input for identifying optical transitions and electronic critical points associated with interband transitions in the P3HT active layer.

The Critical Point (CP) model was implemented in MATLAB R2023b (academic license) using a multi-start nonlinear optimization approach with the Levenberg–Marquardt least-squares solver. This strategy minimizes the likelihood of convergence to suboptimal local minima and ensures reliable extraction of optical parameters. For each critical point, all possible dimensionalities were considered: 0D ($n = -1$), 1D ($n = -0.5$), and 2D ($n = 0$). The CP analysis was performed on the second derivative of the complex dielectric function, ε(E), following the standard formulations [4,7]:

$$\frac{d^2\varepsilon}{dE^2} = \left(\frac{n}{2} - 1\right)\left(\frac{n}{2} - 2\right) A e^{i\emptyset} (E - E_0 + i\Gamma)^{\frac{n}{2} - 3} \qquad (n \neq 2)$$

$$\frac{d^2\varepsilon}{dE^2} = A e^{i\emptyset} (E - E_0 + i\Gamma)^{-2} \qquad (n = 2)$$

where A is the amplitude, φ the phase angle, E_0 the transition energy, and Γ the broadening parameter.

All possible combinations of dimensionalities (0D, 1D, 2D) were considered for each critical point, taken as a fixed discrete integer. The fitting process employed 20 multi-start optimization routines (more than 20 provided no added value) to systematically explore the parameter space and identify the global minimum of the residual function. The quality and robustness of the fits were assessed using the Mean of Squared Errors (MSE) and the 95% confidence intervals for each fitted parameter. The selected dimensionality was therefore determined by comparing the fit quality of the physically defined integer-dimensional CP forms, rather than by interpreting a continuously fitted value of (n_j) as a statistically estimated quantity. These statistical measures ensured the reliability of the extracted morphological and optical descriptors derived from the CP model.

Because the dimensionality selects a discrete line-shape form while leaving the number of free parameters unchanged, information criteria such as AIC and BIC reduce to the residual ranking and add no independent discrimination, and a nested F-test is undefined for these non-nested, equal-parameter forms. We therefore select the dimensionality by exhaustive minimization of the fit residual for every candidate. The continuous parameters carry 95% confidence intervals (Table S3).

No additional background or high-energy oscillators were included within the fitting window. The window 1.7–3.0 eV was chosen because it contains the three P3HT excitonic transitions of interest while excluding spectral regions dominated by other transitions and by increased noise in ε_2. The dimensionality n_j was treated as a discrete integer fit; parameter bounds, initial values, the number of multi-start runs, the convergence tolerance was set to `1e-30` , and the principal parameter correlations are listed in Table S4. The multi-start CP fit of one ε_2 spectrum takes approximately a few minutes on a standard workstation.

The initial guess for the critical point energy was determined from the minimum points of the second derivative of the imaginary dielectric function graph as a function of photon energy.

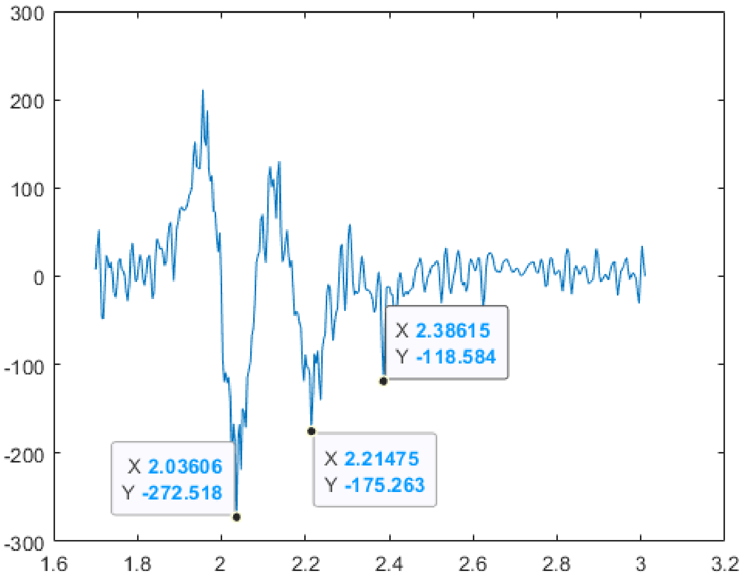

Figure S6. An example of the second derivative of the imaginary dielectric function graph as a function of photon energy for initial guess determination.

Table S3 lists, for each annealing time, the fitted transition energy of each CP with its 95% confidence interval (obtained from the fit covariance matrix), together with the MSE. The confidence intervals are consistent with the transition energies remaining unchanged within uncertainty across annealing time; we note that overlapping intervals do not by themselves prove equivalence. Pearson correlation between CP's parameters and OTFT's current is summarized in Table S4.

Table S3. Confidence intervals and MSE values for each CP's transition energy E_0 and broadening factor as calculated from CP model fitting at various annealing times.

Parameter	Annealing Time (min)	Confidence Interval			MSE
		CP1 ~ 2.03 (eV)	CP2 ~ 2.2 (eV)	CP3 ~ 2.36 (eV)	
E_0	0	±0.005	±0.01	±0.01	0.003
	5	±0.005	±0.006	±0.01	0.002
	10	±0.004	±0.005	±0.02	0.005
	20	±0.006	±0.006	±0.03	0.002
	30	±0.005	±0.008	±0.01	0.002
Γ	0	±0.003	±0.002	±0.001	
	5	±0.003	±0.002	±0.004	
	10	±0.003	±0.002	±0.004	
	20	±0.005	±0.006	±0.007	
	30	±0.007	±0.001	±0.05	

Table S4. Pearson correlation between OTFT's mobility and CP parameters.

Parameter	Pearson R
CP1/CP2 area ratio	0.826
CP1 Gamma	-0.841
CP2 Gamma	-0.852
CP3 Gamma	-0.869
CP1 area	0.838
CP2 area	-0.838
CP3 area	-0.893

References

[1.] Tarekegn, E. N., et al. Photolithographic fabrication of P3HT based organic thin-film transistors with high mobility. *ECS Journal of Solid State Science and Technology*, 2022, 11.2: 025008.

[2.] Xu, Yong, et al. Precise extraction of charge carrier mobility for organic transistors. *Advanced Functional Materials*, 2020, 30.20: 1904508.

[3.] https://www.ossila.com/cdn/shop/files/Example_OFET_test_report.pdf

[4.] HAO, Chu, et al. Experimental determination of short-channel MOSFET parameters. *Solid-State Electronics*, 1985, 28.10: 1025-1030.

[5.] XU, Yong, et al. Direct evaluation of low-field mobility and access resistance in pentacene field-effect transistors. *Journal of Applied Physics*, 2010, 107.11.

[6.] KIM, Gunhee, et al. Extraction of intrinsic contact resistance in organic thin-film transistors with single channel length and high capacitance density. *Applied Physics Letters*, 2021, 119.26.

[7.] H. Fujiwara, Spectroscopic ellipsometry: principles and applications, John Wiley & Sons, 2007.

[8.] R. M. Azzam, N. M. Bashara and S. S. Ballard, Physics Today, 1978.

[9.] Yeh and M. Hendry, Physics Today, 1990.

[10.] Yu, P.Y., and Manuel Cardona. Fundamentals of semiconductors: physics and materials properties. Springer Science & Business Media, 2010.
